# Structure, Functional Properties, and Applications of Foxtail Millet Prolamin: A Review

**DOI:** 10.3390/biom14080913

**Published:** 2024-07-26

**Authors:** Wen Zhang, Guijun Zhang, Wenjing Liang, Jiayi Tian, Shuhao Sun, Xinping Zhang, Xinyi Lv, Peibo Guo, Ao Qu, Zijian Wu

**Affiliations:** 1 Tianjin Key Laboratory of Food Science and Biotechnology, School of Biotechnology and Food Science, Tianjin University of Commerce, Tianjin 300134, China; wener1990@tju.edu.cn (W.Z.); zhangguijun@stumail.tjcu.edu.cn (G.Z.); tianjiayi@stumail.tjcu.edu.cn (J.T.); 120220344@stu.tjcu.edu.cn (X.L.);; 2 Key Laboratory of Low Carbon Cold Chain for Agricultural Products, Ministry of Agriculture and Rural Affairs, Tianjin 300134, China

**Keywords:** foxtail millet prolamin, structure, functional properties, biological activities, application

## Abstract

Foxtail millet prolamin, one of the major protein constituents of foxtail millet, has garnered attention due to its unique amino acid composition and function. Foxtail millet prolamin exhibits specific physicochemical and functional characteristics, such as solubility, surface hydrophobicity, emulsifying, and foaming properties. These characteristics have been exploited in the preparation and development of products, including plant-based alternative products, nutritional supplements, and gluten-free foods. Additionally, because of the favorable biocompatibility and biodegradability, foxtail millet prolamin is frequently used as a carrier for encapsulation and targeted delivery of bioactive substances. Moreover, studies have shown that foxtail millet prolamin is highly nutritious and displays various biological activities like antioxidant effects, anti-inflammatory properties, and anti-diabetic potential, making it a valuable ingredient in medicinal products and contributing to its potential role in therapeutic diets. This review summarizes the current knowledge of the amino acid composition and structural characteristics of foxtail millet prolamin, as well as the functional properties, biological activities, and applications in functional food formulation and drug delivery strategy. Challenges and future perspectives for the utilization of foxtail millet prolamin are also pointed out. This review aims to provide novel ideas and broad prospects for the effective use of foxtail millet prolamin.

## 1. Introduction

Foxtail millet (*Setaria italic* (L.) P. Beaux.) stands out as one of the earliest cultivated crops, deeply rooted in the history of agriculture [1]. It thrives primarily in arid and semi-arid regions, serving as a staple food in various parts of eastern and central Asia. Its global prominence is evident from its remarkable position as the sixth-highest-yielding millet worldwide [2]. This exceptional crop is predominantly grown in China, India, and Japan, and it has characteristics such as a short growth cycle, an impressive average yield of 2166 kg/ha, drought tolerance, resilience, and adaptability [3,4]. In addition, foxtail millet shares nutritional similarities with staple grains such as wheat and rice, encompassing essential components like fiber, minerals, protein, and phytochemicals [5,6]. A comprehensive analysis of its nutritional composition reveals noteworthy properties that foxtail millet contains elevated levels of protein, registering at 11.65%, and its dietary fiber content accounts for 14% of its composition. Notably, it has a relatively low lipid content of 3.48% but a commendable mineral content of 3%. Additionally, foxtail millet contains a diverse array of phytochemicals, including phenolic acids, flavonoids, and β-carotene. Foxtail millet’s gluten-free nature has further contributed to its popularity in the production of gluten-free food products like biscuits and bread, particularly catering to individuals with celiac disease [7]. Furthermore, foxtail millet offers potential health advantages in preventing cancer and cardiovascular disease, assisting in weight loss, and helping reduce the levels of lipids in the blood [8,9]. These properties make foxtail millet an excellent resource for both food and pharmaceutical industries.

**Figure 1:**
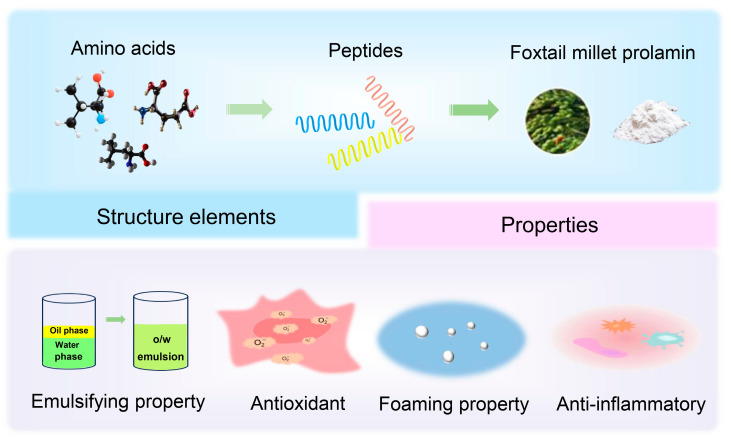
Overview of foxtail millet prolamin.

Foxtail millet, similar to other grains such as wheat, barley, sorghum, and maize, contains a diverse range of proteins, including albumin, globulin, glutelin, and prolamin. Among these, prolamin constitutes the majority (60–65%) of the storage protein in foxtail millet [10]. Prolamin is characterized by its high solubility in alcohol and is distinguished by its elevated levels of hydrophobic amino acids, particularly proline, glutamic acid, valine, and phenylalanine. Millet prolamin is composed of distinct subunits, namely α, β, and γ, which have unique molecular weights and solubility properties [10]. Additionally, both foxtail millet prolamin and peptides derived from prolamin hydrolysate exhibit various physicochemical and functional characteristics that determine their utilization and performance in the food and pharmaceutical industries. This review aims to present an overview of the structure, physicochemical properties, and applications of foxtail millet prolamin, especially the recent advances in functional food formulation, stabilization, and drug delivery strategy (Figure 1). We also discuss the limitations and challenges of the application of foxtail millet prolamin, which will inspire and propel further research and innovation.

## 2. The Structure of Foxtail Millet Prolamin

### 2.1. Amino Acid Composition

The properties of proteins are mainly determined by the types and amounts of amino acids they contain. As investigated by Zhang et al., foxtail millet prolamin consists of 18 different amino acids, among which glutamic acid, leucine, and alanine are the most abundant, accounting for 23.3%, 15.86%, and 9.5% (*w/w*) of the total quantified amino acids, respectively [11]. These results are in line with a previously published work, which reported similar percentages of glutamic acid, leucine, and alanine as 23.67~25.29%, 12.71~13.96%, 9.83~10.24% (*w/w*), respectively [10]. The composition of amino acids plays a crucial role in determining the solubility, palatability, and even nutritional value of foxtail millet prolamin. The composition of acidic and basic amino acids in prolamin varies significantly, and the ratio between them affects the solubility of the protein by influencing the ionization of surface groups [12]. The relatively high levels of non-polar amino acids in prolamin further contribute to its solubility behavior [11]. For the texture and taste profile, the palatability of foxtail millet prolamin is closely related to the proportion of different types of amino acids present [10]. Another characteristic feature of prolamin is its elevated proline content, which enables it to easily bind and interact with phytochemicals, promoting the delivery and absorption of these naturally active substances in the body [11]. Besides, it was found that more than 40% of the amino acids present in the foxtail millet prolamin were essential amino acids, which are crucial for the body’s proper functioning [13]. The high concentration of hydrophobic amino acids in foxtail millet prolamin highlights its importance as a valuable source of essential amino acids [14]. However, it should be noted that the deficiency or insufficient amounts of lysine and tryptophan in foxtail millet prolamin has a detrimental effect on its nutritional value [15].

### 2.2. Folding Blocks

**Figure 2:**
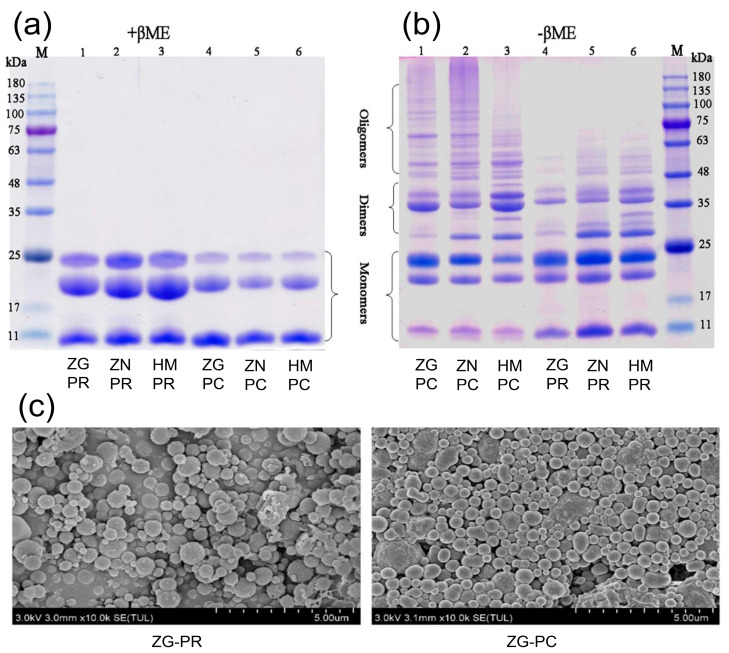
SDS-PAGE patterns and morphology of foxtail millet prolamin. SDS-PAGE patterns of prolamin isolates from three foxtail millet varieties under reducing conditions. (**a**) SDS-PAGE patterns of prolamin isolates from three foxtail millet varieties under non-reducing condition lanes. (**b**) Morphology of prolamin isolates from ZG-PR and ZG-PC varieties at 10 k × magnification. (**c**) PR, prolamin isolates from raw flour; PC, prolamin isolates from cooked flour; ZG, Zhongu variety; ZN, Zhaonong variety; HM, Hongmiao variety.

The local conformation of the protein polypeptide backbone plays a crucial role in determining protein functions and spatial structures [16]. In a specific investigation conducted by Fu et al., prolamin was isolated from defatted foxtail millet flour and followed by analysis using sodium dodecyl sulfate-polyacrylamide gel electrophoresis (SDS-PAGE), which identified three primary peptides with molecular weights of 22, 11, and 24 kDa [17], named α-, β-, and γ-prolamin, respectively. This finding aligns with a previous report that prolamin consists of three to four bands within the molecular weight range of 13–27 kDa [13,18]. Interestingly, the researchers noticed strikingly distinct band patterns when applying non-reducing conditions during the SDS-PAGE analysis of foxtail millet prolamin in comparison to reducing conditions (Figure 2a,b). Under non-reducing conditions, several prolamin dimers ranging from 25 to 48 kDa, as well as trimers and larger oligomers of higher molecular weight (>48 kDa), were observed. However, these bands disappeared under reducing conditions, indicating that all substantial prolamin dimers and oligomers of higher molecular weight (>24 kDa) were linked by disulfide bridges and hydrolyzed into prolamin monomers (11–14 kDa) [10]. Furthermore, it was noted that the predominant secondary structure of foxtail millet prolamin is α-helix, followed by β-turn, which is attributed to the high proportions of hydrophobic residues within the prolamin sequence [10]. The secondary structure of foxtail millet prolamin measured by Fourier transform infrared (FTIR) spectra showed high contents of α-helix and β-turn [10]. Nevertheless, the result contradicted the finding from circular dichroism (CD) spectra, which indicated the presence of α-helices without β structures [19]. The discrepancy in results could be due to different measurement methods, as FTIR spectra are considered more reliable for estimating antiparallel β-sheets and other structures, while CD spectra present higher accuracy in measuring α-helix structures [20]. Further investigations revealed that heat or ultrasonic processing led to a reduction in the proportions of α-helix and random coil structures within the prolamin, accompanied by an increase in β-sheet content, signifying the denaturation and unfolding of foxtail millet prolamin [14,21]. It is worth noting that a high content of β-sheet structures inhibited the accessibility of proteolytic enzymes to the protein substrate, resulting in decreased protein digestibility [22].

### 2.3. Spatial Structure

The protein spatial structure is crucial for understanding the functional properties of proteins [23,24]. To fully comprehend the protein spatial structure, it is essential to visualize the tertiary and quaternary topologies of proteins. As reported by Chen et al., X-ray diffraction measurement was used to examine foxtail millet prolamin’s crystalline structure, where two peaks at the diffraction angles 2 θ of 20° and 9° were detected, but no enhanced diffraction peaks were observed, indicating the amorphous nature of foxtail millet prolamin [25]. Interestingly, prolamin exhibits a unique ability to encapsulate curcumin within nanoparticles, effectively preserving its active amorphous state. This distinctive characteristic highlights the potential of foxtail millet prolamin as a versatile delivery vector for bioactive compounds [26]. Furthermore, foxtail millet prolamin derived from diverse varieties exhibited distinct tertiary structures, while all tryptophan residues were apparent in a polar microenvironment. When prolamin is subjected to heat processing, it experiences changes in its tertiary and quaternary structure, disrupting the overall folding and interactions of the protein resulting in the repositioning of tryptophan residues into a hydrophilic environment [10]. The formation of disulfide bonds, facilitated by the interaction between cysteine residues, plays a pivotal role in shaping the structural and functional characteristics of proteins [27]. Foxtail millet prolamin contains a significant amount of sulfhydryl bonds, which is crucial in stabilizing the assembly of proteins and promoting the formation of multimers, thereby maintaining their functional activity [10]. In addition, the high proportion of hydrophobic amino acids, accompanied by the presence of aromatic amino acids on the molecular surface, contributed significantly to the surface hydrophobicity of foxtail millet prolamin [28]. This characteristic holds implications for the interaction of prolamin with other molecules and surfaces. According to the analysis by scanning electron microscopy (Figure 2c), we can observe that foxtail millet prolamin is in a spherical state [10]. Although some insights into the spatial structure of prolamin have been elucidated, further research is necessary to gain a comprehensive understanding of its characteristic features.

## 3. Characteristics of Foxtail Millet Prolamin

In the preceding section, we outlined the structural properties of foxtail millet prolamin and highlighted their potential implications for the protein’s characteristics. Given that protein properties considerably influence their utility and function, this section aims to provide a thorough examination of the properties inherent to foxtail millet prolamin. These properties include surface charge, solubility, surface hydrophobicity, denaturation temperature, foaming property, emulsifier property, anti-oxidant activities, anti-inflammatory activities, and other biological activities (Table 1). Understanding these properties in depth is crucial for assessing the potential applications of prolamin as components in industrial systems. Despite the significant advancements in elucidating the properties of prolamin, there are ongoing challenges in enhancing these properties through technological interventions. To ensure a thorough comprehension of the diverse characteristics of prolamin, this chapter will be organized into three sections: physicochemical properties, functional properties, and biological properties. By dissecting each aspect separately, readers will gain a comprehensive insight into the multifaceted nature of the prolamin.

### 3.1. Physicochemical Properties

Some physicochemical characteristics of proteins, including molecular size, surface charge, and surface hydrophobicity, have a significant impact on their functional properties, such as solubility, emulsifying, and foaming properties [33]. As for foxtail millet prolamin, the particle size was influenced by high-intensity ultrasound (HIU) treatments and the pH level of the environment. Under specified conditions (ultrasound amplitude: 10%, acoustic power intensity: 37.44 W/cm^2^, treatment time: 20 min, pH: 9), the particle size reached its lowest level at 1.001 μm. This reduction in particle size led to an increased exposed surface area due to the cleavage of hydrophobic bonds, which further facilitated the formation of protein aggregating in the networks [14]. The surface charge of proteins often quantified as Zeta potential, serves as a critical indicator for determining the ionization of surface groups and, ultimately, the solubility of proteins [12]. Prolamin derived from various foxtail millet varieties exhibits Zeta potential values within the range of −18 to −24 mV. Conspicuously, prolamin demonstrates a lower content of non-polar hydrophobic amino acids but a higher surface charge of proteins [10]. Notably, at the isoelectric point (pH = 5), prolamin shows the lowest zeta potential, ranging from −3.0 to −8.6 mV, leading to a negligible charge and the lowest solubility of protein molecules [29]. In contrast to zeta potential, the surface hydrophobicity of prolamin is highest when the pH is closest to the isoelectric point (pH = 5), with a recorded value of 40.12, which is attributed to heightened exposure of hydrophobic amino acid residues [29]. Foxtail millet prolamin exhibits relatively low solubility due to its pronounced hydrophobicity. However, following HIU treatment, the solubility of prolamin significantly increased by 89.58%, 91.35%, 85.23%, and 82.58% at pH 3, 5, 7, and 9, respectively. This remarkable improvement can be attributed to the phenomenon of cavitation, which causes the disruption of hydrogen and hydrophobic bonds within the protein molecules, thereby reducing the molecular weight and augmenting interactions between water molecules and proteins [14]. The water holding capacity (WHC), which refers to the binding capacity of proteins and water against gravity, ranges from 1.87 to 2.30 g/g for foxtail millet prolamin. Interestingly, the WHC differs among different varieties of foxtail millet and is significantly reduced to 0.54–0.93 g/g after cooking. The decrease in WHC affects the utilization of water molecules by starch granules and has an impact on the overall eating quality of foxtail millet [10]. The denaturation temperature serves as a crucial index of the thermal stability of protein. The peak denaturation temperature (Td) of foxtail millet prolamin ranged from 84.68 to 86.24 °C, while the denaturation enthalpy (∆H) ranged from 16.14 to 22.29 J/g. After heat treatment, both Td and ∆H significantly reduced, indicating a decrease in thermal stability compared to the native state of the protein [10]. Moreover, previous studies have substantiated that thermal stability was closely related to the secondary structure of proteins. Especially, an increase in the presence of β-sheet configuration was associated with higher denaturation temperature [34].

### 3.2. Functional Properties

#### 3.2.1. Emulsifying Properties

The emulsifying property of protein at the oil-water interface is a crucial factor in determining their interaction. Two important parameters, the emulsion activity index (EAI) and emulsion stability index (ESI), reflect the ability of proteins to be absorbed in the oil-water interface and remain at the interface after storage, respectively [35]. As previously reported, foxtail millet prolamin exhibited relatively modest emulsification properties, with the EAI ranging from 5.5 to 14.22 m^2^/g and ESI of 27.96~46.02 min [14]. This is in contrast to protein concentrates with higher solubility levels, which have significantly higher EAI and ESI values (EAI = 40.41–75.23 m^2^/g, ESI = 14.88–42.85 min) [14]. The lower emulsifying properties of prolamin can be attributed to its insolubility in aqueous systems and elevated levels of disulfide bonding. These characteristics limit its efficacy to function as an effective emulsifier [36]. However, it has been found that the emulsifying properties of prolamin can be enhanced through HIU treatment, which is mainly due to the unfolding of the globular protein structure and the release of non-polar groups bound within the hydrophobic core [30,37]. As a result, the re-alignment and positioning of the protein molecules at the oil-water interface improves their surface hydrophobic and hydrophilic characteristics, leading to increased emulsification activity. Notably, surface hydrophobicity has been found to have a significant correlation with the emulsifying properties of prolamin, and HIU treatment increases surface hydrophobicity while also decreasing the average particle size, resulting in optimized emulsification performance [14]. Other factors, such as zeta potential and protein content, including both soluble and insoluble fractions, also play a role in protein emulsification properties, similar to the importance of solubility and surface hydrophobicity [38]. However, the influence of other factors, such as pH condition, on emulsification characteristics still requires thorough investigation.

#### 3.2.2. Foaming Properties

Foaming capacity (FC) and foaming stability (FS) are crucial parameters for assessing the capacity of proteins to generate and maintain a foam, which is of paramount importance in food processing. In a recent study conducted by Sharma et al., the FC and FS values of foxtail millet prolamin were found to be 7.32% and 57.89%, respectively, indicating the ability of foxtail millet prolamin to generate and sustain a foam [14]. The foaming properties of prolamin were closely related to its surface hydrophobicity and solubility profiles [39]. After being subjected to HIU treatment, the foaming capacity and foaming stability of prolamin remarkably enhanced compared to the untreated counterparts, with a 20% increase in foaming capacity and a 40% rise in foaming stability. This enhancement can be ascribed to the increased exposure of hydrophobic moieties of the proteins, facilitating absorption at the gas-water interface, thereby improving the formation and sustainability of the foam [30]. Moreover, the foaming characteristics of foxtail millet proteins were further analyzed. It was observed that these proteins exhibit the highest foaming capacity at pH 10, indicating that an alkaline environment is conducive to the formation of long-lasting foam structures. The ability of proteins to form stable foams was intricately related to their water solubility, and proteins with high solubility demonstrated enhanced interactions with water, resulting in preferable bubble-forming capabilities [40].

### 3.3. Biological Activities

#### 3.3.1. Anti-Oxidant Activities

Reactive oxygen species (ROS) and free radicals are harmful substances that directly attack important targets within the body, leading to oxidative damage during metabolic processes [41]. Therefore, it is crucial to maintain appropriately low levels of free radicals and ROS for human health. To counter the damages caused by imbalances of ROS, organisms have developed endogenous antioxidant defenses. Unfortunately, these defenses may sometimes be insufficient, and the intake of antioxidant compounds from food becomes necessary [42]. Extensive research has been conducted on proteins, peptides, and amino acids derived from food, as they possess antioxidant properties by reducing oxidative stress [43,44,45]. In a study conducted by Ji et al., two novel peptides were identified from foxtail millet prolamin that was hydrolyzed by alcalase. These peptides were characterized by the amino acid sequences Pro-Phe-Leu-Phe (PFLF) and Ile-Ala-Leu-Leu-Ile-Pro-Phe (IALLIPF) and exhibited molecular weights of 522.3 Da and 785.5 Da, respectively [19]. Remarkably, these peptides exhibited superior antioxidant activity, as evidenced by their high 1,1-diphenyl-2-picrylhydrazyl (DPPH) radical scavenging activity, reaching up to 149 μM TE/g protein and their oxygen radical absorbance capacity (ORCA) up to 1180 μM TE/g. Furthermore, the antioxidant activity of these two peptides has been investigated in vivo. In a subsequent study by Ji et al., the antioxidant efficacy of three millet prolamin peptides (MPP), including peptides with molecular weights below 1 kDa, PFLF, and IALLIPF, was investigated in human keratinocyte HaCaT cells and RAW264.7 murine macrophages [31]. The result revealed that MPP exhibited notable antioxidant properties, effectively mitigating the production of ROS and malondialdehyde (MDA) while increasing the level of glutathione (GSH), demonstrating excellent anti-oxidant activity in H_2_O_2_-treated HaCaT cells. The researchers also explored the factors that influenced the antioxidant activity of peptides derived from foxtail millet prolamin. It was found that the IALLIPF displayed superior ROS scavenging ability and increased GSH activity compared to the other two peptides, while PFLF showed preferable capacity in reducing MDA. This could be attributed to the differences in the amino acid composition of the peptides. Previous research has elucidated that peptides containing hydrophobic amino acids such as Pro, Leu, Ala, Trp, and Phe had significant antioxidant capacity [46]. Additionally, it is noteworthy that individual amino acids exert varying effects on antioxidant activity. For example, the presence of leucine in peptide sequences enhances the solubility of peptides in lipids, thereby contributing to the inhibition of lipid oxidation. Proline, on the other hand, could impose conformational constraints on peptides, leading to increased antioxidant activity of these amino residues [47].

#### 3.3.2. Anti-Inflammatory Activities

**Figure 3:**
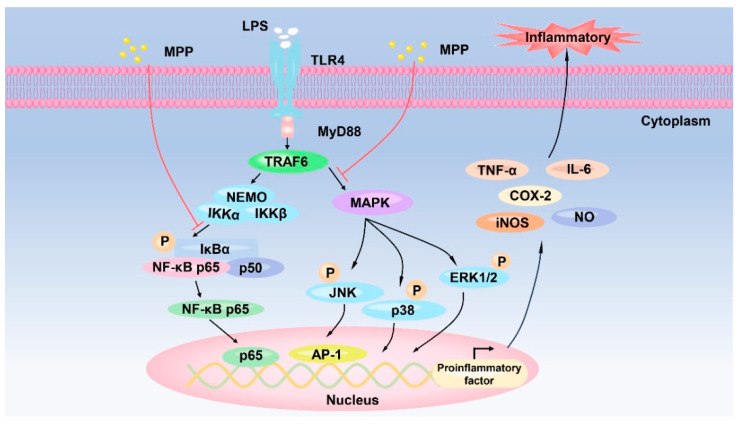
The anti-inflammatory mechanism of MPP on MAPK and NF-κB signal pathway.

Inflammation serves as a fundamental protective response of the human body against external pathogens and endogenous damage [48]. In previous studies, extensive research efforts have been dedicated to elucidating the molecular mechanisms that underlie inflammation. It has been found that the activation of specific pathways, such as the mitogen-activated protein kinase (MAPK) pathway and NF-κB pathway, plays a pivotal role in triggering inflammatory responses [49,50]. Downstream factors, including NF-κB and cyclic AMP-responsive element (CRE), are also implicated in the regulation of inflammation [50,51]. These pathways facilitate the upregulation of immune-related pro-inflammatory factors, such as inducible nitric oxide synthase (iNOS) and cyclooxygenase-2 (COX-2), leading to the accumulation of nitric oxide (NO) and the production of pro-inflammatory factors such as cytokines (e.g., TNF-α and interleukins) and prostaglandins (PGs) [52]. In a particular study, MPP was found to exhibit notable anti-inflammatory properties. The specific mechanism of action is shown in Figure 3.

These peptides, including peptides with a molecular weight below 1 kDa obtained from hydrolysate by alcalase treatment, such as PFLF and IALLIPF, exhibited the ability to inhibit the production of nitric oxide (NO) and pro-inflammatory cytokines (TNF-α, IL-6, and IL-1β) in RAW267.4 murine macrophages when stimulated with lipopolysaccharide (LPS) [31]. Interestingly, it was observed that IALLIPF also demonstrated a therapeutic effect on the regulation of NO and other pro-inflammatory mediators within a specific concentration range. Mechanistically, it was found that MPP regulated the NF-κB pathway by inhibiting the expression of p-IκB and the nuclear translocation of p65 [31]. These findings suggest that MPP may exert its anti-inflammatory effects by impeding the production of phosphorylated proteins in the MAPK and NF-κB pathway.

#### 3.3.3. Other Biological Activities

In addition to the antioxidant and anti-inflammatory properties, hydrolysates or peptides derived from foxtail millet prolamins have been linked to other beneficial effects, including anti-diabetic potential and anti-hypolipidemic effects. Fu et al. demonstrated the potential anti-diabetic activity of prolamin from cooked foxtail millet (PCFM) in diabetic mice by managing glucose homeostasis disorders and alleviating triglyceride accumulation. Peptides obtained from PCFM through pepsin and trypsin hydrolysis exhibited significant α-glucosidase inhibitory activity. Interestingly, cooking enhanced the binding affinity of the peptide sequences towards α-glucosidase, thereby potentially contributing to improved regulation of blood glucose [17]. This finding holds promising implications for the utilization of foxtail millet prolamin in the food industry. Moreover, the aqueous extract of Seteria italica seeds has been shown to have an anti-hyperlipidemic effect in diabetic-treated rats. The extract obviously reduced the levels of triglycerides, low-density lipoprotein (LDL), and very low-density lipoprotein (VLDL) cholesterol while simultaneously increasing the level of high-density lipoprotein (HDL) cholesterol [32]. This indicated that foxtail millet could potentially be used as a natural and effective approach to manage and lower lipid levels in individuals with hyperlipidemia. However, despite these promising findings, further investigation is necessary to fully understand the potential mechanism of lipid-lowering effects of prolamin.

## 4. Applications of Foxtail Millet Prolamin

**Figure 4:**
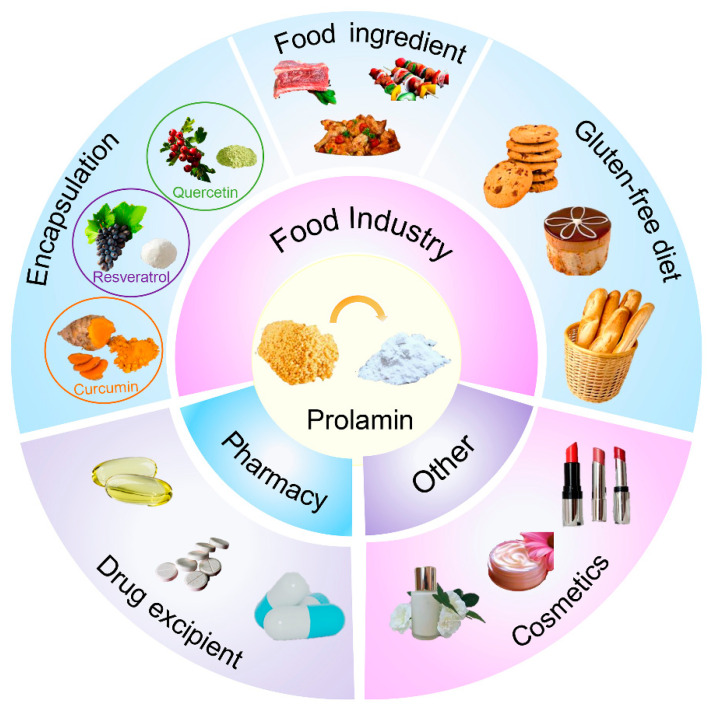
Application of foxtail millet prolamin.

Foxtail millet prolamin has gained significant attention for its potential use in both the food and pharmaceutical industries. Numerous studies have contributed to a comprehensive understanding of its physicochemical properties, structural composition, and sensory characteristics. Foxtail millet prolamin possesses various functional attributes, including solubility, water-holding capacity, foaming, and emulsification properties, as well as antioxidative and anti-inflammatory properties, making it highly desirable for a wide range of applications. In the food industry specifically, foxtail millet prolamin has proven to be a particularly valuable component in the production of gluten-free products, functional food ingredients, and nutritional supplements. In addition, the favorable biocompatibility and biodegradability of foxtail millet prolamin make it an ideal material in delivery systems. Furthermore, the stability and emulsification properties of foxtail millet prolamin enhance its suitability for film and coating applications, such as encapsulating and protecting sensitive ingredients. In this section, the valorization prospects of foxtail millet prolamin in the fields of gluten-free products, food ingredients, encapsulation, and therapeutic products are discussed (Figure 4).

### 4.1. Development of Foxtail Millet Prolamin in Food-Related Fields

#### 4.1.1. Gluten-Free Products

As the number of individuals with gluten sensitivity rises, there is a growing demand for gluten-free foods [53]. Studies have revealed that foxtail millet, which does not contain gluten, is a suitable dietary choice for individuals with celiac disease. The prolamin in foxtail millet is diverse from the protein found in wheat gluten. By incorporating foxtail millet flours into daily diets, it may be possible to delay or even prevent certain pathological conditions, such as celiac disease [54]. While foxtail millet has been successfully utilized in the production of gluten-free products like bread and biscuits, the absence of gluten makes the dough’s structure more fragile, leading to more challenges in improving texture and sensory properties during food preparation [7,55]. Consequently, numerous studies have been conducted to enhance the quality of gluten-free products. One approach is to incorporate multiple additives that mimic the viscoelastic behavior of gluten, which could significantly improve the overall quality and stability of the product [55]. For instance, hydrocolloids such as carboxymethylcellulose (CMC) exhibit exceptional water retention capacity and have the unique ability to form gels at high temperatures, creating a natural barrier membrane in biscuits that reduces water loss and fat penetration [55]. As a result, the sensory characteristics and overall acceptability of gluten-free products are improved. In addition to CMC, the hydrocolloid hydroxypropyl methylcellulose (HPMC) also exhibits a prodigious capacity to enhance the rheological properties of gluten-free foods. It creates a hydrocolloid network within the dough that improves the internal structure, consistency, specific volume, and porosity of bread crumbs [56]. In the study of Abdollahzadeh et al., the combination of concentrated raisin juice (CRJ) and HPMC within a specific concentration range formed new chemical bonds, significantly enhancing the overall quality of gluten-free products [57]. This study provides empirical support for the efficacy of gum additives in enhancing water retention in breadcrumbs compared to pectin. In addition to hydrocolloids, enzymes also play a pivotal role in augmenting the quality of gluten-free bread production. For example, glucose oxidase (GO), xylanase (XYL), and protease (PR) were incorporated into foxtail millet flour to investigate their effect on gluten-free millet bread [7]. The researchers found that the specific volume of bread and the elasticity of breadcrumbs improved significantly after enzyme treatment. Compared to GO and XYL, the PR treatment particularly resulted in reduced hardness, chewiness, and caking properties of breadcrumbs, which received high marks in sensory evaluations. The incorporation of elm bark powder as an additive is another viable strategy for improving the properties of gluten-free bread. This additive enhances gelatinization and rheological characteristics, thereby improving both the specific volume and hardness of the bread [58]. The addition of these hydrocolloids and enzymes to the formulation containing foxtail millet shows promise.

#### 4.1.2. Utilization of Foxtail Millet Prolamin as a Food Ingredient

Prolamin possesses a diverse range of functionalities, including elasticity, foaming, emulsification properties, water binding capacity, and gelation within food matrices, making it highly valuable in the food processing industry. Proteins endowed with superior foaming properties and a high capacity for retaining water play a pivotal role in the production of bread, corn-based products, sausages, and other food items [59]. Recent studies have highlighted the potential health benefits of foxtail millet prolamin as a functional food ingredient. Prolamin-derived peptides, as mentioned above, have been identified to exhibit various beneficial effects, including anti-inflammatory, anti-oxidative, hypoglycemic, and lipid-lowering effects [19]. Subsequent studies have also demonstrated that foxtail millet prolamin peptides could reduce reactive oxygen species and increase glutathione levels, showcasing their antioxidant capacity and extraordinary ability as functional food ingredients [31]. Furthermore, foxtail millet prolamin exhibits promising potential in ameliorating glucose homeostasis disorders, regulating intestinal flora, and modulating serum metabolism, making it suitable for the development of anti-diabetic food products [60]. Fermentation of foxtail millet by microbial strains yields peptides endowed with antioxidant and antimicrobial properties, thus broadening its potential applications in alternative food supplements and preservatives [61]. It is worth noting that foxtail millet prolamin contains a rich array of essential amino acids, making it a valuable nutrient-rich ingredient. With the escalating cost of animal-derived protein, there is a growing interest in natural, clean, and sustainable plant-derived proteins [62,63]. Prolamin, with its abundant essential amino acids, has attracted widespread attention as an unconventional plant-protein source. The versatility of foxtail millet prolamin extends its utilization as a food ingredient in a wide range of dishes, such as soups, stews, and casseroles, where it can be used as a replacement for meat or beans to enhance nutritional content. Sharma et al. treated foxtail millet prolamin with HIU to improve protein properties and expand the potential utilization of millets as a source of plant protein [14]. Furthermore, foxtail millet prolamin can be incorporated into various forms, such as powder, protein bars, or shakes, making it convenient to consume as a nutritional supplement. These supplements support muscle growth and repair, aid in weight management, and serve as a beneficial substitute for conventional protein sources such as soy or wheat, thereby enhancing the nutritional value of food products. Additionally, it can serve as a sustainable and cost-effective plant protein source, addressing the growing demand within the food and feed industries [14]. Overall, foxtail millet prolamin presents the potential as a highly valuable food ingredient for individuals with food sensitivities and various chronic diseases. Its superior biological activities make it a beneficial addition to a well-rounded and healthy diet.

#### 4.1.3. Protein Nanoparticles for Encapsulation

Prolamin, which is rich in nonpolar amino acids, particularly proline, plays a crucial role in interactions with phytochemicals [64]. These interactions promote its ability to encapsulate active substances within the hydrophobic core, providing protection and sustained release of bioactive compounds. Because of its favorable biocompatibility, biodegradability, and strong affinity with bioactive substances, prolamin is frequently used as a delivery system [65]. The applications of prolamin-based nanoparticles for the encapsulation and delivery of food-bioactive compounds are shown in Table 2. The properties of prolamin have sparked interest in utilizing prolamin-based nanoparticles for delivery applications. Curcumin (diferuloylmethane), a bioactive substance derived from Curcuma longa plants, was successfully encapsulated into foxtail millet prolamin (FP)-based nanoparticles through hydrophobic forces and hydrogen bonds [25]. Furthermore, sodium casein (NaCas) served as a stabilizing agent to mitigate the inherent hydrophobic interactions associated with prolamins [66]. The coating of FP-NaCas significantly increased the water solubility of curcumin by an astounding 12400-fold compared to uncoated nanoparticles. The encapsulation strategy not only protected curcumin from degradation but also enhanced its antioxidant activity [25], suggesting the potential of prolamin as an encapsulation agent for preserving and delivering hydrophobic active biomolecules. Additionally, the synthesis method used to create the nanoparticles significantly affects the drug delivery efficacy. Notably, the co-assembled FP-NaCas nanoparticles produced by the pH-cycle method exhibit superior retention of curcumin during long-term storage compared to nanoparticles coated with NaCas using the anti-solvent method [67]. Both types of nanoparticles could be easily dispersed in water, opening up possibilities for their use as functional ingredients in food products. In addition, research has explored the single or co-encapsulation capabilities of foxtail millet prolamin nanoparticles for various hydrophobic active biomolecules. In the study of Chen et al., different bioactive compounds, such as pueraria, resveratrol, diosmetin, and curcumin, were encapsulated within the nanoparticles to explore encapsulation capacity, storage stability, and in vitro release profiles of FP nanoparticle delivery systems [11]. The results revealed that bioactive compounds could be distributed in different hydrophobic regions of the prolamin-based nanoparticles. This phenomenon not only improved the loading efficiency and long-term stability but also highlighted the potential of FP nanoparticles for co-encapsulation of different bioactive substances [68]. Moreover, researchers have elucidated that the encapsulation efficiency (EE) and loading capacity (LC) of FP nanoparticles can be further augmented by using biopolymer complexes. Transport carriers play a crucial role in safeguarding bioactive substances from degradation and ensuring their effective delivery and targeted release in the gastrointestinal tract [69]. The deposition and co-assembly of curdlan sulfate increased the encapsulation efficiency of curcumin, rising from 78.3% to 81.8% and 85.9%, respectively, while slightly reducing the release of curcumin under physiological pH conditions [70]. Similar results were observed when chondroitin sulfate (CS)-sodium caseinate (NaCas)-stabilized FP composite nanoparticles (NPs) were used, with a notably higher curcumin encapsulation efficiency of 93.4% and improved pH stability [66]. Moreover, CS coating exhibited superior inhibition of tumor growth compared to free curcumin and FP-NaCas NP-encapsulated curcumin, suggesting that FP-NaCas-CS NPs have significant advantages for encapsulating hydrophobic drugs, enhancing the efficacy of cancer treatments, and mitigating adverse effects [66]. Additionally, the deposition of chitosan hydrochloride on the co-assembled FP-NaCas complex resulted in smaller particle sizes and higher ionic strength stability compared to NaCas-coated FP NPs [67]. Furthermore, the co-coating of lecithin and sodium alginate on FP nanoparticles exhibited a controlled release of encapsulated quercetin through physical barriers and the ability to enter epithelial cells. By incorporating cryoprotectants such as sucrose, the nanoparticles could be recovered with the same desirable properties, making them highly suitable for industrial applications of natural delivery systems [71]. Overall, nanoencapsulation of bioactive compounds in foxtail millet prolamin offers unique advantages, such as reduced ingredient cost, diminished enzyme digestibility, and improved stability, making it an excellent choice for various delivery applications.

### 4.2. Role of Foxtail Millet Prolamin in the Pharmaceutical Field

Certainly, foxtail millet prolamin exhibits promising applications in the development of medicinal products and therapeutic diets. Numerous studies have confirmed the medical benefits of foxtail millet prolamin in mice and cell models, demonstrating its ability to prevent organismic peroxidation, metabolic disorder, immune dysregulation, hyperglycemia, and hyperlipidemia [17,31]. One specific application of foxtail millet prolamin is in the management of diabetes diseases. The underlying mechanism can be attributed to the reduction of blood glucose levels by inhibiting the generation of α-glucosidase, an enzyme responsible for converting complex dietary carbohydrates into glucose, thus contributing to controlling blood glucose concentration [72]. Apart from its recognized potential in diabetes management, foxtail millet prolamin or its derived peptides possess various functional characteristics such as antioxidant, anti-inflammatory, hypolipidemic effects, and even anticancer activity, which paves the way for the utilization of foxtail millet prolamin as nutraceutical or pharmaceutical components [31]. However, the scarcity of empirical data in this area highlights the need for further research to fully harness and exploit its therapeutic potential. Currently, foxtail millet prolamin is commonly employed as a functional ingredient in pharmaceutical excipients, particularly in tablets and capsules. It has been developed as a stabilizing agent in protein-based drug formulations, as it enhances stability, solubility, and other desirable characteristics. Foxtail millet prolamin has demonstrated the ability to form stable complexes with various pharmaceuticals, indicating its potential to enhance the shelf-life of drugs and protect against detrimental influences from pH fluctuations, temperature changes, and environmental factors. Studies have explored the possibility of using foxtail millet prolamins as carriers for oral drug delivery systems and stabilizers for protein-based drugs and revealed their remarkable ability to form stable complexes with various pharmaceuticals [61,73]. Similar to zein, foxtail millet prolamin may have the potential to be used as a substitute for plastic in biopolymers, presenting a more eco-friendly alternative. However, it is important to acknowledge that the majority of the research on foxtail millet prolamin properties is still in the laboratory setting. Transitioning these findings into commercial applications poses significant challenges, including scalability, regulatory compliance, and economic feasibility. Only through rigorous research and development efforts can the complete potential and benefits of foxtail millet prolamin be realized by the commercial market.

## 5. Challenges and Prospects

In the past two decades, substantial endeavors have been dedicated to investigating and exploring the relationship between the structure, function, and practical application of prolamin derived from foxtail millet. However, the comprehension of prolamin’s intricacies within foxtail millet remains incomplete. In order to fully understand the functional properties and explore the utilization of foxtail millet prolamin, it is crucial to conduct a multifaceted exploration of various methods to modify prolamin. Moreover, prolamin, which is rich in hydrophobic amino acids but lacks basic and acidic counterparts, has film-forming characteristics similar to zein, making it a promising candidate as a natural coating or film material in food preservation. Concurrently, prolamin is a valuable component and sustainable alternative to animal-derived ingredients for developing natural and vegan cosmetics, such as creams, lotions, and makeup products. Studies have indicated that foxtail millet has antioxidant and anti-inflammatory activities, which render it conducive to promoting skin health. On the other hand, as a film-forming agent, prolamin could establish a protective barrier on the skin or hair, augmenting smoothness and fortification. Thus, the presence of foxtail millet prolamin in shampoos, conditioners, and hair masks may enhance their conditioning and moisturizing properties. Despite these potential benefits, the utilization of foxtail millet prolamin in the cosmetic industry is still limited, which highlights the need for further development to fully explore the potential of foxtail millet prolamin in this field.

## 6. Conclusions

The ongoing advancements are providing a deeper understanding of the unique properties of foxtail millet prolamin, allowing for its potential applications across various industries. This review aims to summarize the structure and multifaceted functional properties of foxtail millet prolamin and highlights its potential in the food and pharmaceutical industries. Foxtail millet prolamin has the capability to create nutritious foods with substantial health benefits by acting as a substitute for animal proteins. Its favorable structural properties further make it an ideal candidate for drug delivery systems, opening up new possibilities for the treatment of chronic diseases. While previous studies have offered valuable insights into the structural properties and potential applications of foxtail millet prolamin, there is still a pressing need for further extensive research. By delving deeper into the intricate relationship between structure and function, scientists could uncover novel opportunities for utilizing foxtail millet prolamin in large-scale industrial settings. This emphasizes the ongoing efforts to fully tap into the immense potential of foxtail millet prolamin, ultimately paving the way for innovative solutions in the food and pharmaceutical industries.

## Figures and Tables

**Table 1:** Characteristics of foxtail millet prolamin from different sources.

Source of Seed	Characteristics	Description	References
Dhan Foundation India (Madhya Pradesh, India)	Particle size	The lowest level of 1.001 μm after HIU treatment	[14]
The Chifeng Academy of Agriculture and Animal Science (Chifeng, the Inner Mongolia Autonomous Region, China)	Surface charge (zeta potential)	−18~−24 mV among different foxtail millet variety −3.0~−8.6 mV appeared near the isoelectric point at pH = 5	[10] [29]
The Chifeng Academy of Agriculture and Animal Science (Chifeng, the Inner Mongolia Autonomous Region, China)	Surface hydrophobicity (H_0_)	PR: 6031.63~6728.02 PC: 3996.22~4905.13	[10]
Dhan Foundation India (Madhya Pradesh, India)	Solubility	Poorly soluble in water alone, soluble in aqueous ethanol	[14]
The Chifeng Academy of Agriculture and Animal Science (Chifeng, the Inner Mongolia Autonomous Region, China)	Water holding capacity (WHO)	1.87~2.30 g/g before cooking 0.54~0.93 g/g after cooking	[10]
	Thermal stability	Td = 84.68–86.24 °C ∆H = 16.14~22.29 J/g	
Dhan Foundation India (Madhya Pradesh, India)	Emulsifying property	EAI = 5.5~14.22 m^2^/g ESI = 27.96~46.02 min	[14] [30]
	Foaming property	Foaming capacity value is 7.32% Foaming stability value is 57.89%	
Shanxi province, China	Anti-oxidant activity	DPPH radical scavenging activity is 149 μM TE/g protein ORCA value is 1180 μMTE/g	[19]
Shanxi province, China	Anti-inflammatory activity	–	[31]
Dong fang liang Life Technology Co., Ltd. (Datong, Shanxi province, China)	Anti-diabetic activity	Improve glucose homeostasis disorders; Alleviating triglyceride accumulation; α-glucosidase inhibitory activity	[17]
Tirupati (Andhra Pradesh, India)	Anti-hyperlipidemic effect	–	[32]

**Table 2:** Application of prolamin-based nanoparticles for the encapsulation and delivery of food bioactive compounds and their fabrication methods, loading/encapsulation efficiencies, and properties.

Co-Delivery	Bioactive Compounds	Methods	Loading Capacity (LC) and Encapsulation Efficiency (EE)	Improvement of Properties	References
Lecithin; Alginate	Quercetin	Anti-solvent	LC = 4.4% EE = 95.7%	pH stability; Re-dispersibility; Bio-accessibility; Cell-uptake capacity; Controlled release behavior	[71]
Curdlan sulfate; Sodium caseinate	Curcumin	pH-driven	EE = 85.9%	Sustained release performance; Storage stabilities; Bioactivity	[70]
Sodium caseinate	Puerarian; Resveratrol; Diosmetin; Curcumin	Polarity mediation	LC = 7.4~9.2% EE = 60.7~91.9%	Storage stability; Long-term storage stability; Bioavailability	[11]
Caseinate	Curcumin	Antisolvent/ evaporation	EE = 71.3~98.4%	Prevent the degradation of curcumin during heat treatment; Antioxidant; Anti-tumor	[25]
Caseinate chitosan hydrochloride	Curcumin	Antisolvent pH-driven	EE = 85.6%	pH stability; Sustained and controlled release behavior	[67]

## Data Availability

No new data were created in this study. All the data reported in this review were found in original articles cited in the text.

## References

[1] Meherunnahar M., Hoque M.M., Satter M.A., Ahmed T., Chowdhury R.S., Aziz S. (2024). Effect of cooking characteristics, amino acid consistency, and functional properties of composite noodles made from foxtail millet. Meas. Food.

[2] Saleh A.S.M., Zhang Q., Chen J., Shen Q. (2013). Millet Grains: Nutritional Quality, Processing, and Potential Health Benefits. Compr. Rev. Food Sci. Food Saf..

[3] Sachdev N., Goomer S., Singh L.R. (2021). Foxtail millet: A potential crop to meet future demand scenario for alternative sustainable protein. J. Sci. Food Agric..

[4] Austin D.F. (2006). Fox-tail millets (*Setaria*: *Poaceae*)—Abandoned food in two hemispheres. Econ. Bot..

[5] Zhang M., Xu Y., Xiang J., Zheng B., Yuan Y., Luo D., Fan J. (2021). Comparative evaluation on phenolic profiles, antioxidant properties and α-glucosidase inhibitory effects of different milling fractions of foxtail millet. J. Cereal Sci..

[6] Yang T., Ma S., Liu J., Sun B., Wang X. (2022). Influences of four processing methods on main nutritional components of foxtail millet: A review. Grain Oil Sci. Technol..

[7] Sarabhai S., Tamilselvan T., Prabhasankar P. (2021). Role of enzymes for improvement in gluten-free foxtail millet bread: It’s effect on quality, textural, rheological and pasting properties. LWT.

[8] Gupta N., Srivastava A.K., Pandey V.N. (2012). Biodiversity and Nutraceutical Quality of Some Indian Millets. Proc. Natl. Acad. Sci. India Sect. B Biol. Sci..

[9] Zhang A., Liu X., Wang G., Wang H., Liu J., Zhao W., Zhang Y. (2015). Crude Fat Content and Fatty Acid Profile and Their Correlations in Foxtail Millet. Cereal Chem..

[10] Zhang F., Fu Y., Liu Z., Shen Q. (2021). Comparison of the characteristics of prolamins among foxtail millet varieties with different palatability: Structural, morphological, and physicochemical properties. Int. J. Biol. Macromol..

[11] Chen X., Wu Y.-C., Liu Y., Qian L.-H., Zhang Y.-H., Li H.-J. (2022). Single/co-encapsulation capacity and physicochemical stability of zein and foxtail millet prolamin nanoparticles. Colloids Surf. B Biointerfaces.

[12] Ge J., Sun C.-X., Mata A., Corke H., Gan R.-Y., Fang Y. (2021). Physicochemical and pH-dependent functional properties of proteins isolated from eight traditional Chinese beans. Food Hydrocoll..

[13] Parameswaran K.P., Thayumanavan B. (1995). Homologies between prolamins of different minor millets. Plant Foods Hum. Nutr..

[14] Sharma N., Sahu J.K., Choudhary A., Meenu M., Bansal V. (2023). High intensity ultrasound (HIU)-induced functionalization of foxtail millet protein and its fractions. Food Hydrocoll..

[15] Kumar K.K., Parameswaran K.P. (1998). Characterisation of storage protein from selected varieties of foxtail millet (*Setaria italica* (L) Beauv). J. Sci. Food Agric..

[16] Guo X., Tang X., Zhang M., Ma X., Wang J., Liang H. (2024). New progress in the deep understanding of the biocake layer property: Combined effect of neglected protein secondary structure, morphology, and mechanism. Water Res..

[17] Fu Y., Liu Z., Wang H., Zhang F., Guo S., Shen Q. (2023). Comparison of the generation of α-glucosidase inhibitory peptides derived from prolamins of raw and cooked foxtail millet: In vitro activity, de novo sequencing, and in silico docking. Food Chem..

[18] Marcellino L., Junior C., Gander E. (2002). Characterization of Pearl Millet Prolamins. Protein Pept. Lett..

[19] Ji Z., Feng R., Mao J. (2019). Separation and identification of antioxidant peptides from foxtail millet (*Setaria italica*) prolamins enzymatic hydrolysate. Cereal Chem..

[20] Pribic R., Vanstokkum I.H.M., Chapman D., Haris P.I., Bloemendal M. (1993). Protein Secondary Structure from Fourier Transform Infrared and/or Circular Dichroism Spectra. Anal. Biochem..

[21] Stathopulos P.B., Scholz G.A., Hwang Y.-M., Rumfeldt J.A.O., Lepock J.R., Meiering E.M. (2004). Sonication of proteins causes formation of aggregates that resemble amyloid. Protein Sci..

[22] Long G., Ji Y., Pan H., Sun Z., Li Y., Qin G. (2015). Characterization of Thermal Denaturation Structure and Morphology of Soy Glycinin by FTIR and SEM. Int. J. Food Prop..

[23] Du Q., Li H., Tu M., Wu Z., Zhang T., Liu J., Ding Y., Zeng X., Pan D. (2024). Legume protein fermented by lactic acid bacteria: Specific enzymatic hydrolysis, protein composition, structure, and functional properties. Colloids Surf. B Biointerfaces.

[24] Ochoa-Rivas A., Nava-Valdez Y., Serna-Saldívar S.O., Chuck-Hernández C. (2017). Microwave and Ultrasound to Enhance Protein Extraction from Peanut Flour under Alkaline Conditions: Effects in Yield and Functional Properties of Protein Isolates. Food Bioprocess Technol..

[25] Chen X., Zhang T.-Y., Wu Y.-C., Gong P.-X., Li H.-J. (2022). Foxtail millet prolamin as an effective encapsulant deliver curcumin by fabricating caseinate stabilized composite nanoparticles. Food Chem..

[26] Jog R., Burgess D.J. (2017). Pharmaceutical Amorphous Nanoparticles. J. Pharm. Sci..

[27] Ito K., Inaba K. (2008). The disulfide bond formation (Dsb) system. Curr. Opin. Struct. Biol..

[28] Bamdad F., Wu J., Chen L. (2011). Effects of enzymatic hydrolysis on molecular structure and antioxidant activity of barley hordein. J. Cereal Sci..

[29] Li R., Xiong Y.L. (2021). Ultrasound-induced structural modification and thermal properties of oat protein. LWT.

[30] Jhan F., Gani A., Noor N., Shah A. (2021). Nanoreduction of Millet Proteins: Effect on Structural and Functional Properties. ACS Food Sci. Technol..

[31] Ji Z., Mao J., Chen S., Mao J. (2020). Antioxidant and anti-inflammatory activity of peptides from foxtail millet (*Setaria italica*) prolamins in HaCaT cells and RAW264.7 murine macrophages. Food Biosci..

[32] Sireesha Y., Kasetti R.B., Nabi S.A., Swapna S., Apparao C. (2011). Antihyperglycemic and hypolipidemic activities of Setaria italica seeds in STZ diabetic rats. Pathophysiology.

[33] Karaca A.C., Low N., Nickerson M. (2011). Emulsifying properties of chickpea, faba bean, lentil and pea proteins produced by isoelectric precipitation and salt extraction. Food Res. Int..

[34] Shevkani K., Singh N., Rana J.C., Kaur A. (2014). Relationship between physicochemical and functional properties of amaranth (*Amaranthus hypochondriacus*) protein isolates. Int. J. Food Sci. Technol..

[35] Gani A., ul Ashraf Z., Noor N., Ahmed Wani I. (2022). Ultrasonication as an innovative approach to tailor the apple seed proteins into nanosize: Effect on protein structural and functional properties. Ultrason. Sonochemistry.

[36] Agboola S., Ng D., Mills D. (2005). Characterisation and functional properties of Australian rice protein isolates. J. Cereal Sci..

[37] Dickinson E., Galazka V.B. (1991). Emulsion stabilization by ionic and covalent complexes of β-lactoglobulin with polysaccharides. Food Hydrocoll..

[38] Kaur M., Singh N. (2005). Studies on functional, thermal and pasting properties of flours from different chickpea (*Cicer arietinum* L.) cultivars. Food Chem..

[39] Bejosano F.P., Corke H. (1999). Properties of protein concentrates and hydrolysates from Amaranthus and Buckwheat. Ind. Crops Prod..

[40] Li C., Yang J., Yao L., Qin F., Hou G., Chen B., Jin L., Deng J., Shen Y. (2020). Characterisation, physicochemical and functional properties of protein isolates from Amygdalus pedunculata Pall seeds. Food Chem..

[41] Liochev S.I. (2013). Reactive oxygen species and the free radical theory of aging. Free Radic. Biol. Med..

[42] Wu S., Wang X., Qi W., Guo Q. (2019). Bioactive protein/peptides of flaxseed: A review. Trends Food Sci. Technol..

[43] Wong F.-C., Xiao J., Wang S., Ee K.-Y., Chai T.-T. (2020). Advances on the antioxidant peptides from edible plant sources. Trends Food Sci. Technol..

[44] Wang X., Fu J., Bhullar K.S., Chen B., Liu H., Zhang Y., Wang C., Liu C., Su D., Ma X. (2024). Identification, in silico selection, and mechanistic investigation of antioxidant peptides from corn gluten meal hydrolysate. Food Chem..

[45] Wang S., Zhao M., Fan H., Wu J. (2022). Emerging proteins as precursors of bioactive peptides/hydrolysates with health benefits. Curr. Opin. Food Sci..

[46] Mendis E., Rajapakse N., Kim S.-K. (2005). Antioxidant Properties of a Radical-Scavenging Peptide Purified from Enzymatically Prepared Fish Skin Gelatin Hydrolysate. J. Agric. Food Chem..

[47] Alemán A., Giménez B., Pérez-Santin E., Gómez-Guillén M.C., Montero P. (2011). Contribution of Leu and Hyp residues to antioxidant and ACE-inhibitory activities of peptide sequences isolated from squid gelatin hydrolysate. Food Chem..

[48] Nathan C., Ding A. (2010). Nonresolving Inflammation. Cell.

[49] Saleem S. (2024). Targeting MAPK signaling: A promising approach for treating inflammatory lung disease. Pathol. Res. Pract..

[50] Capece D., Verzella D., Flati I., Arboretto P., Cornice J., Franzoso G. (2022). NF-κB: Blending metabolism, immunity, and inflammation. Trends Immunol..

[51] Tavares L.P., Negreiros-Lima G.L., Lima K.M., E Silva P.M.R., Pinho V., Teixeira M.M., Sousa L.P. (2020). Blame the signaling: Role of cAMP for the resolution of inflammation. Pharmacol. Res..

[52] Zhu B., He H., Hou T. (2018). A Comprehensive Review of Corn Protein-derived Bioactive Peptides: Production, Characterization, Bioactivities, and Transport Pathways. Compr. Rev. Food Sci. Food Saf..

[53] Jhan F., Gani A., Shah A., Ashwar B.A., Bhat N.A., Ganaie T.A. (2021). Gluten-free minor cereals of Himalayan origin: Characterization, nutraceutical potential and utilization as possible anti-diabetic food for growing diabetic population of the world. Food Hydrocoll..

[54] Santini A., Tenore G.C., Novellino E. (2017). Nutraceuticals: A paradigm of proactive medicine. Eur. J. Pharm. Sci..

[55] Reaz A.H., Abedin M.J., Mohammad Abdullah A.T., Satter M.A., Farzana T. (2023). Physicochemical and structural impact of CMC-hydrocolloids on the development of gluten-free foxtail millet biscuits. Heliyon.

[56] Chen X., Gao J., Cao G., Guo S., Lu D., Hu B., Yang Z., Tong Y., Wen C. (2023). The properties of potato gluten-free doughs: Comparative and combined effects of propylene glycol alginate and hydroxypropyl methyl cellulose or flaxseed gum. Int. J. Food Eng..

[57] Abdollahzadeh A., Vazifedoost M., Didar Z., Haddadkhodaprast M.H., Armin M. (2024). Comparison of the effect of hydroxyl propyl methyl cellulose, pectin, and concentrated raisin juice on gluten-free bread based on rice and foxtail millet flour. Food Sci. Nutr..

[58] Li J., Hu S., Xu M., Min F., Yu T., Yuan J., Gao J., Chen H., Wu Y. (2023). Elm (*Ulmus pumila* L.) bark flour as a gluten substitute in gluten-free whole foxtail millet bread. J. Food Sci. Technol..

[59] López D.N., Galante M., Robson M., Boeris V., Spelzini D. (2018). Amaranth, quinoa and chia protein isolates: Physicochemical and structural properties. Int. J. Biol. Macromol..

[60] Fu Y., Yin R., Liu Z., Niu Y., Guo E., Cheng R., Diao X., Xue Y., Shen Q. (2020). Hypoglycemic Effect of Prolamin from Cooked Foxtail Millet (*Setaria italic*) on Streptozotocin-Induced Diabetic Mice. Nutrients.

[61] Sharma N., Niranjan K. (2018). Foxtail millet: Properties, processing, health benefits, and uses. Food Rev. Int..

[62] Wen C., Zhang J., Yao H., Zhou J., Duan Y., Zhang H., Ma H. (2019). Advances in renewable plant-derived protein source: The structure, physicochemical properties affected by ultrasonication. Ultrason. Sonochemistry.

[63] Sahni P., Sharma S. (2023). Quality characteristics, amino acid composition, and bioactive potential of wheat cookies protein-enriched with unconventional legume protein isolates. Qual. Assur. Saf. Crops Foods.

[64] Monteiro P.V., Virupaksha T.K., Rao D.R. (1982). Proteins of Italian millet: Amino acid composition, solubility fractionation and electrophoresis of protein fractions. J. Sci. Food Agric..

[65] Joye I., Julian McClements D. (2016). Biopolymer-Based Delivery Systems: Challenges and Opportunities. Curr. Top. Med. Chem..

[66] Chen X., Wu Y.-C., Gong P.-X., Zhang Y.-H., Li H.-J. (2022). Chondroitin sulfate deposited on foxtail millet prolamin/caseinate nanoparticles to improve physicochemical properties and enhance cancer therapeutic effects. Food Funct..

[67] Chen X., Wu Y.-C., Qian L.-H., Zhang Y.-H., Gong P.-X., Liu W., Li H.-J. (2023). Fabrication of foxtail millet prolamin/caseinate/chitosan hydrochloride composite nanoparticles using antisolvent and pH-driven methods for curcumin delivery. Food Chem..

[68] Chen G., Dong S., Chen Y., Gao Y., Zhang Z., Li S. (2020). Complex coacervation of zein-chitosan via atmospheric cold plasma treatment: Improvement of encapsulation efficiency and dispersion stability. Food Hydrocoll..

[69] Wei Y., Yu Z., Lin K., Sun C., Dai L., Yang S., Mao L., Yuan F., Gao Y. (2019). Fabrication and characterization of resveratrol loaded zein-propylene glycol alginate-rhamnolipid composite nanoparticles: Physicochemical stability, formation mechanism and in vitro digestion. Food Hydrocoll..

[70] Chen X., Yu C., Wang J.-H., Wu Y.-C., Ma Y., Li H.-J. (2023). Fabrication of curcumin−loaded foxtail millet prolamin−based nanoparticle: Impact of curdlan sulfate on the particle properties. Colloids Surf. A Physicochem. Eng. Asp..

[71] Chen X., Wu Y.-C., Gong P.-X., Li H.-J. (2022). Co-assembly of foxtail millet prolamin-lecithin/alginate sodium in citric acid–potassium phosphate buffer for delivery of quercetin. Food Chem..

[72] Lordan S., Smyth T.J., Soler-Vila A., Stanton C., Ross R.P. (2013). The α-amylase and α-glucosidase inhibitory effects of Irish seaweed extracts. Food Chem..

[73] Rana S.S., Tiwari S., Gupta N., Tripathi M.K., Tripathi N., Singh S., Bhagyawant S.S. (2023). Validating the Nutraceutical Significance of Minor Millets by Employing Nutritional–Antinutritional Profiling. Life.

